# Prenatal Concentrations of Polychlorinated Biphenyls, DDE, and DDT and Overweight in Children: A Prospective Birth Cohort Study

**DOI:** 10.1289/ehp.1103862

**Published:** 2011-10-25

**Authors:** Damaskini Valvi, Michelle A. Mendez, David Martinez, Joan O. Grimalt, Maties Torrent, Jordi Sunyer, Martine Vrijheid

**Affiliations:** 1 Centre for Research in Environmental Epidemiology (CREAL), Barcelona, Spain; 2 Hospital del Mar Research Institute (IMIM), Barcelona, Spain; 3 CIBER Epidemiología y Salud Pública (CIBERESP), Barcelona, Spain; 4 Pompeu Fabra University, Barcelona, Spain; 5 Department of Environmental Chemistry, Institute of Environmental Assessment and Water Research (IDÆA-CSIC), Barcelona, Spain; 6 Àrea de Salut de Menorca, IB-SALUT, Menorca, Spain

**Keywords:** body mass index, dichlorodiphenyldichloroethylene (DDE), dichlorodiphenyltrichloroethane (DDT), environmental obesogens, high-fat intakes, obesity, persistent organic pollutants, polychlorinated biphenyls (PCBs)

## Abstract

Background: Recent experimental evidence suggests that prenatal exposure to endocrine-disrupting chemicals (EDCs) may increase postnatal obesity risk and that these effects may be sex or diet dependent.

Objectives: We explored whether prenatal organochlorine compound (OC) concentrations [polychlorinated biphenyls (PCBs), dichlorodiphenyldichloroethylene (DDE), and dichlorodiphenyltrichloroethane (DDT)] were associated with overweight at 6.5 years of age and whether child sex or fat intakes modified these associations.

Methods: We studied 344 children from a Spanish birth cohort established in 1997–1998. Overweight at 6.5 years was defined as a body mass index (BMI) *z*-score ≥ 85th percentile of the World Health Organization reference. Cord blood OC concentrations were measured and treated as categorical variables (tertiles). Children’s diet was assessed by food frequency questionnaire. Relative risks (RRs) were estimated using generalized linear models.

Results: After multivariable adjustment, we found an increased RR of overweight in the third tertile of PCB exposure [RR = 1.70; 95% confidence interval (CI): 1.09, 2.64] and the second tertile of DDE exposure (RR = 1.67; 95% CI: 1.10, 2.55), but no association with DDT exposure in the population overall. Associations between overweight and PCB and DDE concentrations were strongest in girls (*p*-interaction between 0.01 and 0.28); DDT was associated with overweight only in boys. For DDT we observed stronger associations in children with fat intakes at or above compared with below the median, but this interaction was not significant (*p*-interaction > 0.05).

Conclusions: This study suggests that prenatal OC exposures may be associated with overweight in children and that sex and high-fat intake may influence susceptibility.

In recent years there has been increasing concern about the potential role of endocrine-disrupting chemicals (EDCs) in the development of chronic metabolic diseases such as obesity and type 2 diabetes (Casals-Casas and Desvergne 2011). The imbalance between energy intake and energy expenditure is the primary cause of obesity (McAllister et al. 2009). However, there is still uncertainty related to the etiology of the disease, and environmental factors, including early-life exposure to EDCs and maternal smoking, may increase susceptibility to weight gain and obesity (Heindel 2003; Heindel and vom Saal 2009; Porta et al. 2009b). The growing list of chemicals suspected to contribute to obesity in humans includes persistent organic pollutants (POPs) such as polychlorinated biphenyls (PCBs), hexachlorobenzene (HCB), dichlorodiphenyldichloroethylene (DDE), and its precursor dichlorodiphenyltrichloroethane (DDT) (Casals-Casas and Desvergne 2011).

PCBs, HCB, and DDT/DDE are synthetic organochlorine compounds (OCs) whose production and use has been banned (PCBs, HCB) or restricted (DDT) by the Stockholm agreement (Stockholm Convention on Persistent Organic Pollutants 2004). However, the general population is still exposed to these substances at low doses, mainly through the food chain (Porta et al. 2008). These substances are highly resistant to degradation, have lipophilic properties, and accumulate in animal and human fat tissues for many years (Porta et al. 2008). The fetus and newborn have unique routes of early OC exposure via the placenta and breast milk (Barr et al. 2005; Grimalt et al. 2010; Sala et al. 2001); they are potentially more vulnerable than adults to the activity of EDCs because their detoxification and metabolic mechanisms may still be immature (Casals-Casas and Desvergne 2011).

The metabolic effects of OCs have so far not been explored in animal models. However, a growing body of experimental evidence suggests *in utero* exposure to other EDCs, such as bisphenol A, may disrupt homeostatic controls over adipogenesis and energy balance, resulting in weight gain (Casals-Casas and Desvergne 2011; Casals-Casas et al. 2008; Elobeid and Allison 2008; Grün and Blumberg 2009; Newbold et al. 2007; Tabb and Blumberg 2006). Experimental evidence further suggests that these processes may lead to excessive weight gain later in life only in the presence of a high-fat diet (Grün and Blumberg 2006; Somm et al. 2009) and that effects of EDCs on weight gain may be sex specific (Lassiter et al. 2008; Rubin and Soto 2009; Somm et al. 2009).

Few longitudinal studies have examined the association between prenatal OC exposure and obesity in humans (Hatch et al. 2010; Tang-Péronard et al. 2011). One study reported a positive association between intrauterine DDE and PCB exposures and body mass index (BMI) in the first years of life (Verhulst et al. 2009). Other studies have reported an association between prenatal DDE concentrations and BMI early in infancy (Mendez et al. 2011a) and adulthood (Karmaus et al. 2009). The hypothesis that prenatal EDC exposures may increase susceptibility to the effects of a high-fat diet (Grün and Blumberg 2006), so that subjects with both exposures could more easily become obese, has not been examined in humans. Moreover, although earlier observational studies have reported some sex differences in associations between OCs and early life growth (Tang-Péronard et al. 2011), this evidence has been inconsistent.

Thus, our main aims were to evaluate the association between prenatal concentrations of PCBs, DDE, DDT, and overweight in children 6.5 years of age in a prospective birth cohort study and to examine whether sex or high-fat intakes in childhood may modify these relationships. Smink et al. (2008) previously reported a positive association between prenatal HCB concentrations and overweight in this study cohort, but associations with other OCs were not examined.

## Data and Methods

*Study design.* A population-based birth cohort study [the AMICS–INMA project (Menorca Asthma Multicentre Infants Cohort Study– Infancia y Medio Ambiente)] was established on the Spanish Island of Menorca, recruiting women presenting for antenatal care between April 1997 and June 1998 (94% of those eligible, *n* = 482). We followed their children periodically until they reached the age of 6–7 years. The characteristics of the original population have been described elsewhere (Gascon et al. 2011). Women were interviewed at 20 weeks of gestation to collect information about parental sociodemographic characteristics, self-reported maternal prepregnancy weight and height, history of diabetes, parity, and lifestyle factors (e.g., smoking, alcohol consumption) during pregnancy. Exact dates of birth, birth weight, and history of congenital diseases were obtained from medical delivery records, and umbilical cord blood samples (10 mL) were obtained at birth. Blood samples were also collected from a subset of 360 children at age 4 years. We collected breast-feeding information through interviews with the mothers at follow-up visits after birth. At age 6.5 years, mothers completed a short questionnaire on their child’s physical activity and a 96-item food frequency questionnaire (FFQ), previously validated in adults, on their child’s diet in the past year. Information about the FFQ and the methods used for calculating energy and nutrient intakes are described elsewhere (Chatzi et al. 2007). At age 6.5 years, weight and height, without shoes and in light clothing, were measured by specially trained personnel using standard procedures. Informed consent was signed by all mothers. This study was approved by the ethics committee of the Hospital del Mar Research Institute (IMIM) and conducted according to principles of the Declaration of Helsinki (World Medical Association 2008).

*Analysis sample.* A total of 422 of the 482 initially enrolled children had information about weight and height at 6.5 years of age. Mothers of 360 of these children gave informed consent for cord blood extraction, permitting the estimation of prenatal OC concentrations. Sixteen children born prematurely (< 37 weeks of gestation) were excluded from the analysis, because prematurity is linked to different catch-up growth patterns (Euser et al. 2008). In the remaining analysis population (*n* = 344, 71% of the original cohort) there were no children with congenital diseases.

*Study variables.* Our main study outcome was overweight. We used BMI (weight in kilograms/height in square meters) at 6.5 years of age to classify children as being overweight. To define overweight (including obesity; hereafter described as “overweight”), we compared results using the BMI cutoffs proposed by the World Health Organization (WHO) (de Onis et al. 2007) (based on age-and sex-specific 85th percentiles in the 1977 U.S. National Center for Health Statistics sample) and by the International Obesity Taskforce (IOTF) (Cole et al. 2000) (age-and sex-specific centile curves based on a multicountry sample that at age 18 years pass through the adult cutoff point of 25 kg/m² for adult overweight). Because the magnitude and the direction of associations were the same using either of these two references (not shown), we present only the results obtained using the WHO cutoffs. We also analyzed BMI as continuous outcome variable, using age-and sex-specific BMI z-scores according to the WHO reference (de Onis et al. 2007).

Prenatal concentrations of PCBs, DDE, DDT, and HCB were estimated in cord blood. The samples were analyzed by gas chromatography coupled to electron capture detection (Hewlett Packard 6890N GC-ECD; Avondale, PA, USA) carried out in the Department of Environmental Chemistry (IDÆA-CSIC) in Barcelona, Catalonia, Spain, as described elsewhere (Grimalt et al. 2010). The total PCB concentration was determined by summing PCB congeners 28, 52, 101, 118, 138, 153, and 180. The limits of detection (LOD) and quantification (LOQ) for all OCs ranged between 0.007 and 0.323 and 0.011 and 0.485, respectively, and between 5% and 100% of the samples were > LOQ value (Table 1). Samples < LOD were set to half the LOD, and concentrations between the LOD and LOQ were set to the midpoint between the LOD and LOQ. In 216 subjects, OC levels were also measured in blood samples of the children at 4 years of age using the same methods. The total lipid content in cord blood was determined gravimetrically in a subgroup (*n* = 92) of the original population [mean total lipids = 2.6 g/L; 95% confidence interval (CI): 1.2, 5.6]. Because lipid content information was not available for all samples, OC concentrations used in analyses were not lipid corrected.

**Table 1:** Average cord blood OC concentrations, LOD and LOQ values, and percentage of quantifiable samples in the analysis population (*n* = 344).

OC (ng/mL)	LOD (ng/mL)	LOQ (ng/mL)	% > LOQ	Geometric mean (GSD)
PCBs								0.75 (1.70)
PCB-28		0.112		0.178		5		0.07 (1.56)
PCB-52		0.069		0.108		11		0.05 (1.76)
PCB-101		0.037		0.056		28		0.04 (2.10)
PCB-118		0.030		0.046		76		0.06 (2.26)
PCB-138		0.031		0.046		95		0.14 (2.01)
PCB-153		0.023		0.034		98		0.18 (1.92)
PCB-180		0.008		0.012		95		0.13 (2.82)
DDE		0.026		0.041		100		1.06 (2.45)
DDT		0.007		0.011		93		0.08 (3.81)
HCB		0.323		0.485		73		0.67 (1.95)
GSD, geometric standard deviation.								

Children’s fat intakes were calculated from the FFQ, and relative fat intakes were derived as the percentage of daily energy intake from fat. Because energy and food intakes assessed via FFQs are prone to bias (Hill and Davies 2001), we identified implausible diet reports by calculating for every child the ratio of reported energy intake to predicted energy needs, as suggested elsewhere (Huang et al. 2005). This method identified 3.2% as underreporters and 30.5% as overreporters.

*Statistical analyses.* We applied generalized additive models (GAMs) to explore the shape of the relationships between PCB, DDE, and DDT concentrations and overweight. These models did not always indicate clear linear relationships (p-gain defined as the difference in normalized deviance between the GAM model and the linear model for the same predictor < 0.05). Thus, concentrations were modeled as categorical variables (tertiles). Generalized linear models (Zou 2004) were used to estimate the relative risk (RR) of overweight in relation to exposure. Continuous BMI *z*-scores were analyzed using multivariate linear regression. We evaluated as possible confounders a large number of risk factors for childhood obesity and predictors of OC concentrations from the literature: sex, exact age at follow-up, gestational age at birth, birth weight, breast-feeding duration, child’s daily energy intake (in kilocalories), and relative intakes of fat, protein, carbohydrate (as percentages of energy intake), child’s intake of fruits and vegetables and sugar-sweetened beverages (in grams per kilocalorie) at age 6.5 years, sleep duration, hours spent watching television per week, time spent in sedentary activities (including television watching, studying, using a computer, and playing videogames: ≤ 1 hr/day, 2 hr/day, ≥ 3 hr/day), daily time spent in sports after school (< 0.5, ≥ 0.5 hr), maternal age, parity, maternal pregnancy BMI, maternal history of diabetes, maternal alcohol consumption and smoking during pregnancy, and maternal and paternal education and social class. Social class was defined using the U.K. Registrar General 1990 Classification based on occupations coded following the ISCO 88 (Warwick Institute for Employment Research 2004). We classified professional/managerial/technical occupations as nonmanual; skilled, partially skilled, and unskilled occupations as manual; and unemployed and housewives as unclassified. We retained covariates in the final models if they modified any of the coefficients of interest by > 10%. Accordingly, all final models were adjusted for continuous birth weight, maternal pregnancy BMI (at or below vs. above 25 kg/m²), smoking in pregnancy (no/yes), parity (nulliparous no/yes), maternal education (secondary education completed no/yes) and social class, maternal age (< 30, 30–35, > 35 years), and breast-feeding duration (< 2, 2–25, > 25 weeks). Data were complete for all covariates except maternal and paternal education and maternal prepregnancy BMI, which were missing for only 1–4% of observations [see Supplemental Material, Table 1 (http://dx.doi.org/10.1289/ehp.1103862)]. Because exclusion of subjects with missing values could lead to loss of precision, we performed multiple imputation of missing covariates by generating 20 imputed data sets, the results of which were combined to produce estimates and CIs that incorporated missing data uncertainty (Donders et al. 2006).

We studied the associations of interest separately for each OC and in a multipollutant model including PCBs, DDE, DDT, and HCB. Spearman correlations between pollutants [rho (*p*-value)] were 0.40 (< 0.01) for PCBs–DDE, 0.27 (< 0.01) for PCBs–HCB, –0.02 (0.62) for PCBs–DDT, 0.41 (< 0.01) for DDE–HCB, 0.41 (< 0.01) for DDE–DDT, and 0.29 (< 0.01) for DDT–HCB.

Heterogeneity in associations related to child sex or children’s fat intakes were evaluated by including interaction terms in the final models and by stratifying the sample according to sex or fat intakes at or above versus below the median value of 35.7% of energy intake. Models adjusted for dietary variables and models stratifying by fat intake were adjusted for misreporting (under, over vs. plausible diet reporters), because previous studies have found such adjustments to be influential in analyses of FFQ-reported dietary factors (Mendez et al. 2011b).

Because children with low or high birth weight may have a different obesity risk later in life and because size at birth may have been influenced by intrauterine exposures, we conducted sensitivity analyses restricting the sample to children in the normal range of weight at birth (2,500–4,000 g; *n* = 318) (Oken and Gillman 2003; Wang et al. 2009; Wojtyniak et al. 2010). As a sensitivity analysis we also assessed the effect of excluding those assumed to be diet misreporters. We further evaluated the effect of postnatal OC exposures on the associations of interest in the subgroup of 216 children with postnatal OC estimates. We also performed a complete case analysis restricted to subjects with no missing data (*n* = 331).

For all comparisons, the statistical significance level was set at *p* < 0.05. All statistical analyses were performed using the statistical package STATA 10.1 (StataCorp, College Station, TX, USA).

## Results

Children included in the analysis (*n* = 344) were similar to those excluded (because of missing exposure or outcome data or because of preterm birth, *n* = 138) with respect to most characteristics, except for a higher prevalence of girls (51.7% vs. 40.6%) [see Supplemental Material, Table 1 (http://dx.doi.org/10.1289/ehp.1103862)]. The prevalence of overweight at 6.5 years was 26.7%: 25.3% among girls and 28.3% among boys. Overweight children had higher birth weight, and their mothers were more likely to have a pregnancy BMI > 25 kg/m² and to have smoked during pregnancy. No other covariates were statistically significantly associated with overweight in the bivariate analysis (Table 2). Fat, protein, and carbohydrate intakes (percentage of energy intake), estimated based on maternally reported food intakes, were similar among non-overweight and overweight children, both in the full sample (*n* = 344) (Table 2) and among plausible diet reporters only (*n* = 228) (not shown). The highest OC concentrations were found for DDE, followed by PCBs, HCB, and DDT (Table 1). Mean cord blood OC concentrations and other characteristics were similar between boys and girls (not shown).

**Table 2:** Characteristics of 344 children and their parents by overweight at 6.5 years.*^a^*

Overweight		
Characteristics	No (*n* = 252)	Yes (*n* = 92)	*p*-Value*b*
Child characteristics						
BMI *z*-score at 6.5 years		–0.03 ± 0.75		1.60 ± 0.37		< 0.01
Sex (female)		133 (52.8)		45 (48.9)		0.52
Age (years)		6.7 ± 0.2		6.7 ± 0.2		0.97
Gestational age (weeks)		39.5 ± 1.2		39.6 ± 1.1		0.96
Birth weight (g)		3,210 ± 441		3,325 ± 424		0.03
Breast-feeding (weeks)						
< 2		49 (19.4)		15 (16.3)		
2–25		118 (46.8)		52 (56.5)		
> 25		85 (33.7)		25 (27.2)		0.28
Fat intake (% of daily EI) at 6.5 years		36.0 ± 3.6		36.0 ± 3.4		0.98
Protein intake (% of daily EI) at 6.5 years		17.2 ± 4.9		17.1 ± 4.9		0.88
Carbohydrate intake (% of daily EI) at 6.5 years		50.3 ± 13.8		50.7 ± 16.5		0.83
Sleep duration at 6.5 years (hr/day)		10.4 ± 0.7		10.3 ± 0.6		0.13
TV watching at 6.5 years (hr/week)		7.5 ± 4.2		7.9 ± 4.6		0.48
Maternal characteristics						
Age at pregnancy (years)						
< 30		138 (54.8)		43 (46.7)		
30–35		84 (33.3)		35 (38.1)		
> 35		30 (11.9)		14 (15.2)		0.40
Parity (nulliparous)		123 (48.8)		43 (46.7)		0.73
Prepregnancy BMI > 25 kg/m²		39 (15.7)		29 (31.9)		< 0.01
Smoking any time in pregnancy (yes)		83 (32.9)		41 (44.6)		0.04
Alcohol consumption in pregnancy (yes)		20 (7.9)		10 (10.9)		0.39
Diabetes (yes)		15 (5.9)		5 (5.4)		0.85
Education more than secondary		106 (43.3)		33 (38.4)		0.45
Social class						
Nonmanual		120 (47.6)		36 (39.1)		
Manual		80 (31.7)		37 (40.2)		
Unclassified		52 (20.6)		19 (20.6)		0.29
Paternal characteristics						
Education more than secondary		81 (32.8)		37 (41.1)		0.16
Social class						
Nonmanual		85 (33.7)		33 (35.9)		
Manual		162 (64.3)		57 (61.9)		
Unclassified		5 (2.0)		2 (2.2)		0.93
EI, energy intake. Overweight defined as a BMI *z*-score ≥ 85th percentile of the WHO reference standard (de Onis et al. 2007).**a**Values are mean ± SD for continuous and *n* (%) for categorical variables. **b**Chi-square for categorical variables; *t*-test (for normally distributed) or Wilcoxon–Mann–Whitney (for non-normal distribution) for continuous variables.						

**Figure 1:**
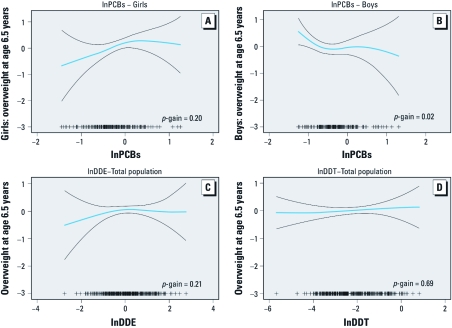
Generalized additive models of the adjusted*ª* associations of prenatal lnPCB*^b^* for girls (*n *= 178) (*A*) and boys (*n *= 166) (*B*), and lnDDE(*n *= 343)*^c^* (*C*) and lnDDT (*n *= 344) (*D*) concentrations*^d^* (nanograms per milliliter) with overweight at 6.5 years. Abbreviations: ++, number of observations; *p*-gain, difference in fit between GAM and linear models. Blue line is the smoothed function for (ln)OCs and black lines are the 95% CIs. ***ª***Adjusted for birth weight, previous parity, maternal prepregnancy BMI, maternal education and social class at pregnancy, maternal smoking in pregnancy, maternal age at delivery, and breast-feeding. ***^b^***Presented separately in girls and boys because of statistically significant sex interaction. ***^c^***One influential outlier excluded: the observation with the highest DDE exposure (lnDDE = 2.97 ng/mL). ***^d^***Log-transformed because distributions were skewed to the right.

GAMs examining the shape of the relationships between overweight and OC concentrations (Figure 1) showed that these were linear for DDT (*p*-gain = 0.69), but less clearly so for DDE (*p*-gain = 0.21) and PCBs (*p*-gain = 0.06) in the population as a whole. For PCBs, nonlinearity was seen in boys (*p*-gain = 0.02) rather than girls (*p*-gain = 0.20).

Cord blood PCB concentrations in the third tertile (> 0.9 ng/mL) compared with the first tertile (< 0.6 ng/mL) were positively and significantly associated with overweight at 6.5 years after multivariable adjustment (RR = 1.70; 95% CI: 1.09, 2.64); the second tertile of PCB exposure was not associated with overweight (Table 3). The association related to the third tertile was weaker and not statistically significant when adjusted for other OCs (RR = 1.54; 95% CI: 0.95, 2.49) (Table 3). Overweight showed a stronger positive association with PCBs in the highest versus lowest tertile in girls (adjusted RR = 2.13; 95% CI: 0.99, 4.57) than in boys (adjusted RR = 1.43; 95% CI: 0.82, 2.48) (*p*-interaction for the third tertile = 0.07), whereas the association with PCBs in the second tertile was positive for girls (adjusted RR = 1.64; 95% CI: 0.72, 3.76) and inverse for boys (adjusted RR = 0.50; 95% CI: 0.26, 0.97) (*p*-interaction for the second tertile = 0.01) (Table 4). For PCBs, there was no evidence for effect modification by fat intake (Table 4).

**Table 3:** Crude and adjusted estimated effects (RR, 95% CI) of prenatal PCB, DDE, and DDT concentrations on overweight at 6.5 years in the analysis population (*n *= 344).

OC concentrations (ng/mL)	*n*	Crude model	Multivariable-adjusted model*a*	Multipollutant-adjusted model*a,b*
PCBs								
< 0.6 (T1)		110		Reference		Reference		Reference
0.6–0.9 (T2)		117		0.82 (0.51, 1.32)		0.97 (0.58, 1.62)		0.92 (0.54, 1.56)
> 0.9 (T3)		117		1.30 (0.86, 1.96)		1.70 (1.09, 2.64)		1.54 (0.95, 2.49)
DDE								
< 0.7 (T1)		113		Reference		Reference		Reference
0.7–1.5 (T2)		116		1.52 (0.97, 2.40)		1.67 (1.10, 2.55)		1.45 (0.93, 2.24)
> 1.5 (T3)		115		1.41 (0.88, 2.25)		1.28 (0.81, 2.03)		0.94 (0.58, 1.54)
DDT								
< 0.06 (T1)		108		Reference		Reference		Reference
0.06–0.18 (T2)		124		1.32 (0.84, 2.10)		1.19 (0.76, 1.87)		1.12 (0.73, 1.71)
> 0.18 (T3)		112		1.42 (0.90, 2.26)		1.17 (0.73, 1.88)		1.11 (0.68, 1.81)
**a**Adjusted for birth weight, previous parity, maternal prepregnancy BMI, maternal education and social class at pregnancy, maternal smoking in pregnancy, maternal age at delivery, and breast-feeding. **b**Additionally adjusted for HCB and the other OCs shown in this table [all OCs in tertiles (T)].								

**Table 4:** Effects of prenatal PCB, DDE and DDT concentrations on overweight at 6.5 years in the total population and in subgroups, according to child sex or fat intakes.

OC exposure level	PCBs			DDE			DDT		
	Subgroup	*n*	RR*a* (95% CI)	*n*	RR*a* (95% CI)	*n*	RR*a* (95% CI)
Overall (*n* = 344)		T2*b*		118		0.97 (0.58, 1.62)		116		1.67 (1.10, 2.55)		124		1.19 (0.76, 1.87)
		T3*b*		117		1.70 (1.09, 2.64)		115		1.28 (0.81, 2.03)		112		1.17 (0.73, 1.88)
Girls (*n* = 178)		T2*b*		62		1.64 (0.72, 3.76)		61		2.64 (1.18, 5.90)		69		0.90 (0.47, 1.73)
		T3*b*		64		2.13 (0.99, 4.57)		58		1.86 (0.75, 4.63)		57		1.16 (0.59, 2.28)
Boys (*n* = 166)		T2*b*		56		0.50 (0.26, 0.97)		55		1.27 (0.69, 2.34)		55		1.96 (1.06, 3.62)
		T3*b*		53		1.43 (0.82, 2.48)		57		1.31 (0.73, 2.38)		55		1.49 (0.73, 3 .05)
*p* Sex interaction*c*		T2				0.01				0.07				0.10
		T3				0.07				0.28				0.48
Low fat intake (*n* = 172)		T2*b*		53		0.72 (0.36, 1.48)		56		1.65 (0.89, 3.04)		60		1.01 (0.55, 1.85)
		T3*b*		63		1.44 (0.81, 2.56)		52		1.17 (0.60, 2.29)		60		1.08 (0.56, 2.09)
High fat intake (*n* = 172)		T2*b*		65		1.23 (0.58, 2.59)		60		1.89 (0.92, 3.86)		64		2.31 (1.16, 4.58)
		T3*b*		54		1.95 (1.00, 3.82)		63		1.38 (0.72, 2.66)		52		1.62 (0.78, 3.37)
*p* Fat intake interaction*c*		T2				0.37				0.72				0.14
		T3				0.46				0.77				0.41
**a**Multivariable model adjusted for birth weight, previous parity, maternal prepregnancy BMI, maternal education and social class at pregnancy, maternal smoking in pregnancy, maternal age at delivery, and breast-feeding. Stratified models by fat intakes additionally adjusted for diet misreporting. **b**Second (T2) or third tertile (T3) compared with first. **c**Tertile-specific *p* Wald test for interaction.														

For DDE, a statistically significant association with overweight was observed for the second tertile (0.7–1.5 ng/mL) (adjusted RR = 1.67; 95% CI: 1.10, 2.55), but not the third tertile (> 1.5 ng/mL) (Table 3). This association was weaker and not statistically significant after adjustment for the other OCs. The RRs in both the second and third tertiles were somewhat greater in girls (second tertile adjusted RR = 2.64: 95% CI: 1.18, 5.90 and third tertile adjusted RR = 1.86; 95% CI: 0.75, 4.63) than in boys (second tertile adjusted RR = 1.27; 95% CI: 0.69, 2.34 and third tertile adjusted RR = 1.31; 95% CI: 0.73, 2.38) (*p*-interaction = 0.07 for the second and 0.28 for the third tertile) (Table 4). We did not observe any evidence of effect modification by fat intake for DDE (Table 4).

For DDT, we observed no association between prenatal concentrations and overweight in the population overall (Table 3). The second tertile of DDT exposure was significantly associated with overweight in boys (adjusted RR = 1.96; 95% CI: 1.06, 3.62) but not in girls (adjusted RR = 0.90; 95% CI: 0.47, 1.73) (*p*-interaction for the second tertile = 0.10) (Table 4). Among children with higher fat intakes, positive associations were observed in the second (adjusted RR = 2.31; 95% CI: 1.16, 4.58) and third tertile (adjusted RR = 1.62; 95% CI: 0.78, 3.37). In contrast, associations were null among children with low fat intakes. This interaction was not statistically significant (*p*-interaction = 0.14 for the second and 0.41 for the third tertile) (Table 4).

For all three OCs, multipollutant models (not shown) showed similar interaction results to those shown in Table 4. When we used continuous BMI *z*-scores as outcome variable (not shown), we obtained associations that were similar in direction and significance level to those obtained using overweight, for all three OCs, both in the total population and in sex- or fat intake–specific strata. Furthermore, sensitivity analyses restricting the sample to children of normal birth weight or to plausible diet reporters, as well as the complete case analysis, showed no meaningful differences in the strength or direction of these associations (not shown). Finally, in the subgroup of 216 children with postnatal OC concentrations, we obtained similar multivariable-adjusted risk estimates, and these risk estimates remained unchanged after further adjustment for postnatal OC exposure [see Supplemental Material, Table 2 (http://dx.doi.org/10.1289/ehp.1103862)].

## Discussion

This study suggests that prenatal OC exposures are associated with overweight and higher BMI *z*-scores at 6.5 years of age. Associations between overweight and PCB and DDE exposure were stronger in girls than in boys; associations with DDT were seen only in boys. For DDT there was some suggestive evidence for stronger associations in children with fat intakes at or above the median, but the interaction was not significant.

The environmental obesogen hypothesis postulates that EDCs, potentially via inappropriate receptor activation and epigenetic changes, may interfere with adipocyte differentiation and lipid metabolism or storage and thus predispose an organism to obesity, especially if exposure occurs during vulnerable windows of fetal and child development (Casals-Casas and Desvergne 2011; Elobeid and Allison 2008; Grün and Blumberg 2009; Hatch et al. 2010; Heindel 2003; Heindel and vom Saal 2009; Newbold et al. 2007). Numerous animal studies suggest that prenatal exposure to certain EDCs is associated with subsequent increases in body weight (Casals-Casas and Desvergne 2011; Casals-Casas et al. 2008; Elobeid and Allison 2008; Grün and Blumberg 2009; Newbold et al. 2007; Rubin and Soto 2009; Somm et al. 2009; Tabb and Blumberg 2006). Intrauterine xenoestrogen exposures may increase the number and/or size of adipocytes in early life, thereby predisposing individuals to obesity later in life, particularly if they consume a high-fat diet (Grün and Blumberg 2006). The specific effects of OCs on metabolic disruption have not yet been explored in animal models. However, the OCs studied here exhibit estrogenic, antiestrogenic, or antiandrogenic activity, and especially PCBs may alter thyroid function metabolism (Diamanti-Kandarakis et al. 2009). These characteristics led us to hypothesize a potential effect of OCs on weight homeostasis. Mechanisms that might explain the observed associations require further exploration in laboratory studies.

We found a statistically significant positive association between prenatal PCB exposure and childhood overweight in girls only. An earlier study reported a positive association between prenatal PCB exposure and weight adjusted for height in adolescent girls but not boys (Gladen et al. 2000), which is consistent with our results. However, in a different Spanish cohort (*n* = 518), Mendez et al. (2011a) found no association between prenatal PCB concentrations and BMI at 14 months and do not report sex interactions for PCBs; however, PCB concentrations were lower than in our study. Similarly, Karmaus et al. (2009) reported no association between estimated prenatal PCB exposure (as Aroclor 1260; interquintile range 0.05–7.08 ng/mL) and BMI in female adults. Differences in the age at which BMI was assessed may explain inconsistencies, at least partially, between our results and those in other cohorts. Most prospective studies in this field suggest null associations between prenatal PCB exposure and weight, weight adjusted for height, or BMI in boys (Tang-Péronard et al. 2011). To our knowledge, there is no study other than ours suggesting an inverse association between PCBs and overweight in boys with moderate concentrations. EDC effects on weight homeostasis, especially if mediated by dysregulation of sexual hormone receptors, may be sex specific (Grün and Blumberg 2009). PCBs may exhibit estrogenic, antiestrogenic, and antiandrogenic effects (Bonefeld-Jørgensen et al. 2001); thus, there may be differences in the sensitivity to these PCB effects between the two sexes because of differences in the natural androgen–estrogen balance during critical windows of fetal development. Larger studies are needed to confirm the sex differences observed here.

We found a statistically significant association between overweight and the second but not third tertile of DDE exposure that was somewhat stronger in girls than in boys. A recent study in a population with mean DDE levels equal to 125 ng/g lipid also reported a positive association between prenatal DDE concentrations and children’s BMI at age 14 months (Mendez et al. 2011a). DDE concentrations in this other study were substantially lower than our estimated average of 620 ng/g lipid (calculated by using mean lipid levels measured in a small subgroup of children). Concentrations among subjects in our second tertile (269–576 ng/g lipid) were somewhat more comparable with those of subjects at the highest level of exposure in the earlier study (> 186 ng/g lipid). In contrast to our findings, in the study by Mendez et al. (2011a), associations were somewhat stronger in boys than in girls, although sex differences were not statistically significant. Another study, where mean DDE levels were lower (212 ng/g lipid) than in our population, reported a weak positive association between prenatal DDE concentrations and BMI in the first 3 years of life (Verhulst et al. 2009); sex differences were not reported because of modest sample size (*n* = 138). Karmaus et al. (2009) also showed prenatal DDE exposure to be positively associated with BMI in adult women in a cohort with levels much higher than those observed here (interquintile range 1.50–9.40 ng/mL vs. 0.50–2.16 ng/mL in our study). The stronger association we observed for the second rather than the third tertile of DDE concentrations (overall and in girls) may appear counterintuitive, but similar nonmonotonic relationships between low-level POP exposure and obesity have also been reported in adults (Lee et al. 2010, 2011). For example, a recent nested case–control study suggested that compared with higher levels of exposure, moderate levels of DDE and of other POPs, including some PCB congeners, were more strongly associated with increases in BMI 18 years later (Lee et al. 2011). Moreover, experimental evidence on other EDCs suggests that low-level prenatal exposure may have stronger or opposite endocrine effects than higher-level exposure (Arsenescu et al. 2008; Lassiter et al. 2008; Rubin and Soto 2009).

DDT was associated with overweight only in boys; similar to DDE, the association appeared to be somewhat stronger in the second than in the third tertile of DDT exposure (in boys). DDT is an estrogen agonist, whereas its metabolite DDE is an androgen antagonist (Diamanti-Kandarakis et al. 2009). Both estrogenic and antiandrogenic activity play a key role in adipogenesis during development (Bluin et al. 2009; Cooke and Naaz 2004). We speculate that DDE and DDT may have different obesogenic effects in boys and girls because of their different endocrine activity. To our knowledge, association between DDT and BMI has been estimated in only one other study; in a cohort of African-American boys 10–20 years of age (*n* = 304) prenatally exposed to DDT levels, no association was found with BMI or other anthropometric measurements at puberty (Gladen et al. 2004), but the reference group was much more highly exposed (< 900 ng/g lipid) than the reference group defined here (< 23 ng/g lipid).

This is, to our knowledge, the first human study that has evaluated the potential modifying effect of fat intake on the relationship between prenatal EDC exposure and later obesity. Lassiter et al. (2008) observed that compared with controls fed a normal diet, female rats placed on a high-fat diet had enhanced weight gain associated with low-dose neonatal exposure to parathion. Somm et al. (2009) reported body weight increase due to bisphenol A exposure in male rats when animals were placed on a high-fat diet. Although interaction tests did not reach significance, we did find some suggestion in this population that DDT may be related to overweight only among children consuming a high-fat diet. Even though we identified implausible diet reporters to address possible error or bias in estimated intakes, the validity and precision of dietary covariates is somewhat uncertain. Modification by diet as well as the influence of sex on these relationships should be further explored in larger populations.

One limitation of this study is the modest sample size, which limited our capacity to detect statistically significant associations, particularly interactions. Despite some loss to follow-up, children included in the analyses had similar characteristics from those excluded, and thus selection bias is expected to be minimal. The analysis population did have an increased proportion of girls compared with the excluded subjects. However, as there were no important differences in the main characteristics of girls or boys included compared with those excluded (not shown), the probability that the higher prevalence of girls in the included sample may have biased the results is very small. We measured cord blood lipid concentrations only in a subgroup of children (*n* = 90), and thus OC concentrations were not lipid adjusted. However, there is no consensus on whether OC concentrations should be lipid adjusted, especially when samples are obtained from healthy subjects with no important weight losses in past months (Porta et al. 2009a; Schisterman et al. 2005). Finally, in the subgroup of 216 children with postnatal OC estimates, associations were similar before and after adjustment for postnatal OC exposure, suggesting that effects of prenatal exposures were not confounded by postnatal ones.

Multivariate-adjusted associations between PCBs and overweight were substantially strengthened after adjusting for birth weight (third tertile crude RR = 1.30; 95% CI: 0.86, 1.96; and third tertile RR after adjustment for birth weight = 1.50; 95% CI: 0.94, 2.14), although DDE and DDT results were not. As children with low or high birth weights may have a different obesity risk later in life (Oken and Gillman 2003; Wang et al. 2009) and birth weight may have been influenced by prenatal OC exposures (Verhulst et al. 2009; Wojtyniak et al. 2010), it is unclear whether birth weight should be considered an intermediate variable or is best treated as a confounder. Our analyses restricting to normal birth weight children did not change findings, but future larger studies should include test associations in more refined strata according to birth weight.

This study is strengthened by its prospective design and the relatively long follow-up period. Menorca is a rural and suburban site with some agricultural activity and no important sources of OC production (Grimalt et al. 2010). Thus, this population is likely to be representative of nonindustrial populations that follow a general Mediterranean diet pattern.

## Conclusions

This study suggests that prenatal PCB, DDE, and DDT exposures may be associated with overweight in children and that sex and high-fat intake may influence susceptibility. Further epidemiological research in larger populations is needed to assess the consistency of the patterns of association observed here.

## Supplemental Material

(29 KB) PDF
