# Supplementary material for: Prenatal Concentrations of Polychlorinated Biphenyls, DDE, and DDT and Overweight in Children: A Prospective Birth Cohort Study
Source: Environ Health Perspect. 2011 Oct 25;120(3):451–7. doi: 10.1289/ehp.1103862 (PMC3295349; doi:10.1289/ehp.1103862)
Supplement: (29 KB) PDF [file ehp.1103862.s001.pdf]

SUPPLEMENTAL MATERIAL

Prenatal Concentrations of PCBs, DDE, DDT and Overweight in Children: A Prospective Birth Cohort Study

Damaskini Valvi[1,2,3,4,*], Michelle A. Mendez[1,2,3], David Martinez[1,2,3], Joan O. Grimalt[5], Maties Torrent[6], Jordi Sunyer[1,2,3,4], Martine Vrijheid[1,2,3]

[1]Centre for Research in Environmental Epidemiology (CREAL), Barcelona, Spain

[2]Hospital del Mar Research Institute (IMIM), Barcelona, Spain

[3]CIBER Epidemiología y Salud Pública (CIBERESP), Barcelona, Spain

[4]Pompeu Fabra University, Barcelona, Spain

[5]Department of Environmental Chemistry, Institute of Environmental Assessment and Water Research (IDAEA-CSIC), Barcelona, Spain

[6]Àrea de Salut de Menorca, IB-SALUT, Menorca, Spain

[*]Corresponding author contact information: dvalvi@creal.cat

Table Of Contents

Suppl. Material, Table 1. Characteristics in the subsample of excluded subjects and in the analysis sample…………………………………………………..…………….*page 2*

Suppl. Material, Table 2. Multivariable-adjusted estimated effects in the subgroup of children with postnatal OC concentrations…………………………………..……*page 3*

Supplemental Material, Table 1. Comparison of characteristics in the subsample of excluded subjects (*N=138*) and in the analysis sample (*N=344*)

Characteristics	Excluded Subjects (N=138)		Included Subjects (N=344)		
	Value[a]	Missings N (%)	Value[a]	Missings N (%)	p-value[b]
**Child characteristics**					
Sex – Female	56 (40.6)	0 (0%)	178 (51.7)	0 (0%)	0.03
Age – years	6.7 ± 0.2	0 (0%)	6.7 ± 0.2	0 (0%)	0.81
Gestational age – weeks	38.6 ± 2.6[c]	0 (0%)	39.6 ± 1.2	0 (0%)	< 0.01
Birth weight – grams	3052 ± 611[c]	0 (0%)	3240 ± 438	0 (0%)	< 0.01
Breastfeeding – weeks					
<2	34 (24.6)		64 (18.6)		
2-25	72 (52.2)		170 (49.4)		
>25	32 (23.2)	0 (0%)	110 (32.0)	0 (0%)	0.11
**Maternal characteristics**					
Age at pregnancy - years					
<30	67 (56.5)		181 (52.6)		
30-35	42 (30.4)		119 (34.6)		
>35 years	18 (13.0)	0 (0%)	44 (12.8)	0 (0%)	0.67
Parity - nulliparous	71 (51.4)	0 (0%)	166 (48.3)	0 (0%)	0.53
BMI before pregnancy > 25 kg/m²	28 (20.3)	7 (5%)	68 (20.0)	5 (1%)	0.82
Smoking any time in pregnancy – yes	58 (42.0)	0 (0%)	124 (36.1)	0 (0%)	0.22
Alcohol consumption in pregnancy - yes	14 (10.1)	0 (0%)	30 (8.7)	0 (0%)	0.63
Diabetes – yes	5 (3.6)	0 (0%)	20 (5.8)	0 (0%)	0.33
Education - more than secondary	55 (40.7)	3 (2%)	139 (41.9)	13 (4%)	0.80
Social class					
Non-manuals	53 (38.4)		156 (45.3)		
Manuals	48 (34.8)		117 (34.0)		
Unclassified	37 (26.8)	0 (0%)	71 (20.6)	0 (0%)	0.25
**Paternal characteristics**					
Education - more than secondary	38 (28.4)	4 (3%)	118 (35.0)	7 (2%)	0.17
Social class					
Non-manuals	49 (35.5)		118 (34.3)		
Manuals	83 (60.1)		219 (63.7)		
Unclassified	6 (4.3)	0 (0%)	7 (2.1)	0 (0%)	0.34

[a] Values are means ± SD for continuous and N (%) for categorical variables.
[b] Chi-square for categorical variables; t-test (for normally distributed) or Wilcoxon-Mann-Whitney (for non-normal distribution) for continuous variables.
[c] Excluded subjects include preterm births. Excluded subjects after omitting preterm births (N=122); birthweight mean ± SD = 3189 ± 490, p-value=0.23 and gestational age mean ± SD=39.5 ± 1.32, p-value=0.71.

Supplemental Material, Table 2. Adjusted estimated effects (RR, 95% CI) of prenatal PCB, DDE and DDT concentrations on overweight at 6.5 years in the subgroup of children with postnatal OC exposures measured in child's serum at age 4 years (*N=216*)[a].

Prenatal OC concentrations (ng/mL)	N	Multivariable-adjusted model 1[b]	Multivariable-adjusted model 1+ postnatal OC exposure [b,c,d]
**PCBs**			
< 0.6	57	Ref.	Ref.
0.6-0.9	76	1.17 (0.60, 2.27)	1.17 (0.61, 2.27)
> 0.9	83	2.00 (1.12, 3.57)	2.16 (1.22, 3.84)
**DDE**			
< 0.7	65	Ref.	Ref.
0.7-1.5	79	1.70 (0.93, 3.10)	1.71 (0.93, 3.12)
> 1.5	72	1.60 (0.89, 2.92)	1.64 (0.88, 3.06)
**DDT**			
< 0.06	84	Ref.	Ref.
0.06-0.18	73	0.98 (0.55, 1.74)	0.99 (0.55, 1.77)
> 0.18	59	1.06 (0.61, 1.86)	1.06 (0.59, 1.90)

[a] Non-selective subgroup of the analysis population for child characteristics such as sex, age, birthweight, breastfeeding, BMI z-scores, or maternal characteristics such as age, parity, pre-pregnancy BMI, smoking in pregnancy, education and social class.
[b]Adjusted for birthweight, previous parity, maternal pre-pregnancy BMI, maternal education and social class at pregnancy, maternal smoking in pregnancy, maternal age at delivery and breastfeeding.
[c]Additionally adjusted for postnatal exposure of the OC tested (all OCs in tertiles).
[d] Spearman correlations between pre- and post-natal exposures; Rho (p-value)= 0.28 (<0.01) for PCBs, 0.46 (<0.01) for DDE and 0.18 (<0.01) for DDT.
